# Supplementary material for: Service-policy gaps in the settlement journey of Arabic-speaking immigrant newcomer and refugee older adults in Edmonton, Canada
Source: PLoS One. 2024 Oct 18;19(10):e0311646. doi: 10.1371/journal.pone.0311646 (PMC11488703; doi:10.1371/journal.pone.0311646)
Supplement: Attachment — Submitted filename: Rebutal Letter.docx [file pone.0311646.s001.docx]

**Response to reviewers' comments**

Please use the space provided to explain your answers to the questions above. You may also include additional comments for the author, including concerns about dual publication, research ethics, or publication ethics. (Please upload your review as an attachment if it exceeds 20,000 characters)

Dear Editor and Reviewers,

Thank you for your time and efforts in reviewing our manuscript. We greatly appreciate your feedback, which has helped enhance the readability and quality of the paper. We have addressed all of your comments and made the necessary revisions.

Thank you once again for your valuable input.

When submitting your revision, we need you to address these additional requirements.

1. Please ensure that your manuscript meets PLOS ONE's style requirements, including those for file naming. The PLOS ONE style templates can be found at

<https://journals.plos.org/plosone/s/file?id=wjVg/PLOSOne_formatting_sample_main_body.pdf> and

<https://journals.plos.org/plosone/s/file?id=ba62/PLOSOne_formatting_sample_title_authors_affiliations.pdf>

Thank you! We have ensured it.

2. We suggest you thoroughly copyedit your manuscript for language usage, spelling, and grammar. If you do not know anyone who can help you do this, you may wish to consider employing a professional scientific editing service.

The American Journal Experts (AJE) (<https://www.aje.com/>) is one such service that has extensive experience helping authors meet PLOS guidelines and can provide language editing, translation, manuscript formatting, and figure formatting to ensure your manuscript meets our submission guidelines. Please note that having the manuscript copyedited by AJE or any other editing services does not guarantee selection for peer review or acceptance for publication.

Upon resubmission, please provide the following:

The name of the colleague or the details of the professional service that edited your manuscript

A copy of your manuscript showing your changes by either highlighting them or using track changes (uploaded as a *supporting information* file)

All edits have been carefully reviewed and completed by the authors, including those with expertise in research and strong writing skills.

A clean copy of the edited manuscript (uploaded as the new *manuscript* file

3. When completing the data availability statement of the submission form, you indicated that you will make your data available on acceptance. We strongly recommend all authors decide on a data sharing plan before acceptance, as the process can be lengthy and hold up publication timelines. Please note that, though access restrictions are acceptable now, your entire data will need to be made freely accessible if your manuscript is accepted for publication. This policy applies to all data except where public deposition would breach compliance with the protocol approved by your research ethics board. If you are unable to adhere to our open data policy, please kindly revise your statement to explain your reasoning and we will seek the editor's input on an exemption. Please be assured that, once you have provided your new statement, the assessment of your exemption will not hold up the peer review process.

Sure, we will include it. Thank you

4. Please include your full ethics statement in the ‘Methods’ section of your manuscript file. In your statement, please include the full name of the IRB or ethics committee who approved or waived your study, as well as whether or not you obtained informed written or verbal consent. If consent was waived for your study, please include this information in your statement as well.

Thank you for highlighting. We have included these details in the manuscript.

5. Please include captions for your Supporting Information files at the end of your manuscript, and update any in-text citations to match accordingly. Please see our Supporting Information guidelines for more information: <http://journals.plos.org/plosone/s/supporting-information>.

Thank you! We have included this detail at the end of the manuscript.

Reviewer #1: Would liked to have seen how trustworthiness of the data was assured, i.e., credibility, transferability, dependability and confirmability. Describing these methods will strengthen the paper. Overall and interesting paper highlighting important concerns of older Arabic speaking INRs in Canada. I was under the wrong impression that Canada had less resettlement challenges than the US.

Thank you so much for your comment. We have included these details in the manuscript.

Reviewer #2: This interesting paper presents an important qualitative study exploring the challenges experienced by Arabic-speaking immigrant/refugee older adults in Edmonton, Canada during settlement. The paper discusses policy and service implications. The topic of the study will be of value for public health specialists and health/social care providers. Congratulations to the authors. I am offering some suggestions for consideration before publication.

We really appreciate your thoughts on this.

Title
1. Please specify the location; that the study was among Arabic-speaking immigrant/refugee older adults in Edmonton, Canada

Thanks for highlighting this. We have included these details in the title.

Abstract
2. Like comment above, kindly specify location (i.e., Edmonton). This will help the reader gain insight into the exact location/province in Canada where the study was conducted.

Thank you. We have made changes as per your suggestions.

3. Please briefly mention eligibility criteria.

Thank you we have added these details in the abstract briefly.


Introduction

1. Minor typo: in sentence “…. to live within the past five years [3],” please replace comma with period before the following sentence.

Thank you for your insights. We have made changes as per your comments.

2. For sentence “It resettled 30,087 refugees [4].” Do the author mean between 2018-2019? If so, please connect to the previous sentence.

Thank you for your highlighting. We have made it clear.

3. The authors state “Just over 1.3 million new immigrants settled permanently in Canada from 2016 to 2021….”. Does this number include refugees?

Thank you for your highlighting. We have made the necessary changes.

4. Same question for “By 2036, immigrants will represent up to 30% of Canada's population (5)”. Does this number involve refugees?. Also, kindly change the reference from (5) to [5] to be consistent with reference style used.

Thank you for your input, we have addressed this concern.

5. For “Immigration accounts for almost 100% of Canada's labour force growth”, please add reference.

Thank you! This comment has been addressed.

6. The authors state that “They share many beliefs, traditions, and values, particularly concerning the care and support of older persons”. Please give a few examples of these traditions, values.

Thank you for highlighting. We have included additional details.

7. It is thoughtful that the authors mention some of the challenges faced by INR older adults (e.g., language barriers, social isolation, distrust of social services and financial precocity). It would be insightful to please add some of the distinct barriers that Arabic speaking INR older adults may face to highlight the importance of conducting this study with this population.

Thank you for your comment. We have addressed this issue.

Methods
8. By “the researcher team”, do the authors mean “the research team”? If so, please revise for clarity.

Thanks we have made corrections.

9. The authors state “The majority were women, younger than 75 years of age and self-identified as having low income and limited fluency in English. Most were receiving some form of financial support from the Canadian Government”. If possible, please briefly give specific numbers/percentage so the reader can gain some insight when

Thank you for your comment. We have included the number of participants to make it clear.

10. Thank you for outlining the details about the interviews. As per methods section, I understand that 11 interviews (10 individual and 1 with couple) were conducted. Would you please check the abstract and clarify/address any typo in this regard?
11. Would you please give a brief insight into how many interviews were done in Arabic?

Thank you for your comment. We have clarified. These were semi-structured interviews with 12 individuals and 1 with the couple.

Discussion
12. Please briefly discuss findings on food insecurity and how this aligns with findings from other Canadian research, as this is a significant problem in Canada. You may find this recent study in Ontario, Canada helpful (<https://doi.org/10.1016/j.appet.2024.107226>).

Thank you for your suggestion. We have addressed this concern.

13. The authors state “Other major barriers reported by participants were scarcity of culturally appropriate services and limited understanding by service providers of the needs of older migrants.”. Please briefly discuss this important finding, as lack of culturally sensitive services is a significant problem for Arabic speaking immigrants/refugees. You may find this paper helpful
<https://doi.org/10.1007/s00127-024-02668-4>.

Thank you for your suggestion. We have addressed this concern.

14. Please add a brief strengths/limitations section for your thoughtful paper.

Thank you so much for your suggestion. We have included these details.

15. Please add a brief conclusion section.

Thank you so much for your suggestion. We have included these details.
