# Supplementary material for: Service-policy gaps in the settlement journey of Arabic-speaking immigrant newcomer and refugee older adults in Edmonton, Canada
Source: PLoS One. 2024 Oct 18;19(10):e0311646. doi: 10.1371/journal.pone.0311646 (PMC11488703; doi:10.1371/journal.pone.0311646)
Supplement: Attachment — Submitted filename: Rebuttal Letter-19-09-2024.docx [file pone.0311646.s002.docx]

**Response to Reviewer comments:**

Most critiques have been addressed in this revised manuscript. However, it is not clear how the revision addresses the issue of trustworthiness of the data (credibility, transferability, dependability, and confirmability; per Reviewer 1). Please provide an explanation and point to specific part/s of the manuscript where this information is presented.

Dear Reviewer,

Thank you so much for your feedback on the manuscript. We have addressed and made the component of trustworthiness clear. Your comments are valuable in enhancing the quality of our paper.

Thanks
